# THE URBAN AGING RESIDENT’S COALITION: A CASE STUDY IN LATE-ADULTHOOD COMMUNITY ACTIVISM

**DOI:** 10.1093/geroni/igad104.1610

**Published:** 2023-12-21

**Authors:** Kathy Wright, Pamela Shields, Karen Moss, Katie White, Judith Tate, Karen Rose

**Affiliations:** The Ohio State University, Columbus, Ohio, United States; African American Men’s Wellness Organization, Columbus, Ohio, United States; The Ohio State University, Columbus, Ohio, United States; Central Ohio Area Agency on Aging, Columbus, Ohio, United States; The Ohio State University, Columbus, Ohio, United States; The Ohio State University, Columbus, Ohio, United States

## Abstract

The COVID-19 global pandemic, police brutality, and threats to voting rights retraumatized African American older adults, who lived during civil rights, causing feelings of powerlessness. Older adults can be equipped to regain agency and control through community activism. We provide a case study of an older African American activist’s (Ms. P) journey to create the Urban Aging Resident’s Coalition (UARC)—an affiliation of the National African American Men’s Wellness Agency. UARC was founded in 2019 to improve mental and physical health for urban older adults. Post-pandemic, Ms. P along with teenage volunteers, held monthly educational sessions that included interactions with local police and voting rights education. To facilitate a non-confrontational experience, Ms. P had the police bring Golden Retriever therapy dogs and a community support person. To increase relatability for voter education, Ms. P formed a panel of retired legislators, the first African American state senator who developed the Ohio Office of Minority Health, a younger state representative, and the former CEO of the Urban league to hold an open forum on voting rights and redistricting. Nearly 60 older adults attended the monthly educational sessions. The community activist addressed those traumas experienced by older adults in a safe and encouraging social environment. Participants reported feeling more comfortable interacting with the police. Older adults increased their awareness of the importance of voting to elect leaders that represent them and their ideas. This model, to engage minoritized groups to regain agency and power, can be a template for other communities.

